# Bringing our best selves to work: Proactive vitality management and strengths use predicting daily engagement in interaction

**DOI:** 10.3389/fpsyg.2022.1015397

**Published:** 2022-12-15

**Authors:** Zselyke Pap, Delia Vîrgă, Daria Lupșa

**Affiliations:** Department of Psychology, West University of Timișoara, Timișoara, Romania

**Keywords:** proactive vitality management, strength use, work engagement, diary study, JD-R theory

## Abstract

The present research focused on bottom-up, proactive employee behaviors and personal resources that can contribute to more engagement and optimal functioning at work. Based on the Job Demands-Resources (JD-R) and Conservation of Resources (COR) theories, we tested direct and interactive relationships between strengths use (SU), daily proactive vitality management (PVM), and daily work engagement (WE). Eighty-seven (*N* = 87) employees from a multinational company completed self-reported questionnaires at the beginning of the study and throughout five consecutive workdays (*N* = 358), yielding a multilevel dataset. We have found a significant daily positive relationship between PVM and WE, which showed significant inter-individual variation and was significantly enhanced by SU at the individual level. This study showed that PVM as employee-initiated proactive behavior and SU as a proactive personal resource facilitate engagement independently but yield the strongest results when used together, suggesting an interactive mechanism between bottom-up effects postulated in the JD-R theory.

## 1.Introduction

Predicting employee engagement has had tremendous success in the past, mostly driven by the Job Demands-Resources (JD-R) theory (Bakker and Demerouti, 2017). Work engagement (WE) entails experiencing the work as an activity that individuals want to “devote time and effort to” (vigor), perceive as being “significant and meaningful” (dedication), and carry out “fully concentrated and engrossed in it” (absorption; Bakker, 2014, p. 2). Research guided by the JD-R theory showed that organizations need to enrich the resources employees rely on in order to be fully engaged (Bakker and Demerouti, 2017). However, technological developments (i.e., virtual work) and changes in the nature of work (i.e., remote work) have raised the issue that employees need to take more responsibility for their work outcomes and progress (Op den Kamp et al., 2018). This has put the employee center stage, creating a need for research that highlights how employees are active and proactive in changing their work and themselves each day to perform their job (Bakker, 2015; Bakker and Demerouti, 2018).

In line with recent developments in the JD-R theory (Demerouti et al., 2019), this study investigates employee-initiated behavioral strategies in the form of proactive vitality management (PVM; Op den Kamp et al., 2018) as antecedents of daily WE. PVM is defined as “goal-oriented behavior aimed at managing physical and mental energy to promote optimal functioning at work” (Op den Kamp et al., 2018, p. 10). PVM includes self-initiated and goal-oriented behaviors that involve generating energy resources proactively instead of reacting to already depleted energy after periods of work (Op den Kamp et al., 2018). In this sense, it is a distinct concept from related ones, such as recovery experiences (e.g., relaxing and recovering through leisure activities; Sonnentag and Fritz, 2007) and micro-breaks (e.g., surfing the internet, discussing with colleagues; Fritz et al., 2011) which are meant to restore energy and attention that has been already depleted. Recent research has generated enthusiasm toward PVM since initial investigations linked it to higher creativity (Op den Kamp et al., 2020), entrepreneurial performance (Tisu and Vîrgă, 2022), and task performance (Bakker, 2017). Based on Bakker et al. (2020) work, we argue that employees who proactively build energy, inspiration, and motivation at work, consciously managing their own energetic, affective, and cognitive resources during the day, can facilitate their daily WE.

Research has also highlighted significant person-level moderators that can shape the efficiency of such proactive energy management strategies (e.g., goal orientation, Bakker et al., 2020; self-insight, Op den Kamp et al., 2020). Inspired by this literature, we propose that strengths use (SU) could constitute a person-level moderator of the daily relationship between PVM and WE. SU represents employees’ initiative to use their strengths more often to complete work (van Woerkom et al., 2016). Strengths are trait-like characteristics (Wood et al., 2011), and SU can be conceptualized as a personal resource, representing a dispositional or habitual behavior (Kira et al., 2010) to use one’s strengths to perform at work. From the perspective of the Conservation of Resources theory (COR; Hobfoll, 2011), SU as a personal resource can help employees create and/or attract other resources into a resource caravan (Chu et al., 2022; Ding and Lin, 2020). Empirical work has supported this assertion, showing that SU is connected to higher self-efficacy (Meyers and van Woerkom, 2017), person-job fit (Kooij et al., 2017), and positive affect (Meyers and van Woerkom, 2017). SU could represent a possible facilitator of the positive effects of PVM (Op den Kamp et al., 2018) due to its’ potential to create resourceful conditions for “can do” (through feelings of efficacy), “reason to” (through alignment of the job with ones’ values and skills), and “energized to” (through positive affect) motivational states that prompt, proactive goal generation and aid self-regulation in striving to achieve those goals (Parker et al., 2010). This conceptualization of SU as a personal resource being dispositional and habitual implies stability in the construct, representing a person-level characteristic that varies across employees. PVM, on the other hand, is strongly tied to the responsibilities and workflow of any specific day, making it a variable that we can expect to show significant daily variability within employees (Bakker et al., 2020). Adopting a diary design and building a multilevel model whereby intra-individual variation in PVM represents the first level of analysis (Level 1) and inter-individual differences in SU represent the second level (Level 2) of analysis allows us to gauge a complex interaction between more stable and more fluctuating proactive initiatives.

**Figure 1:**
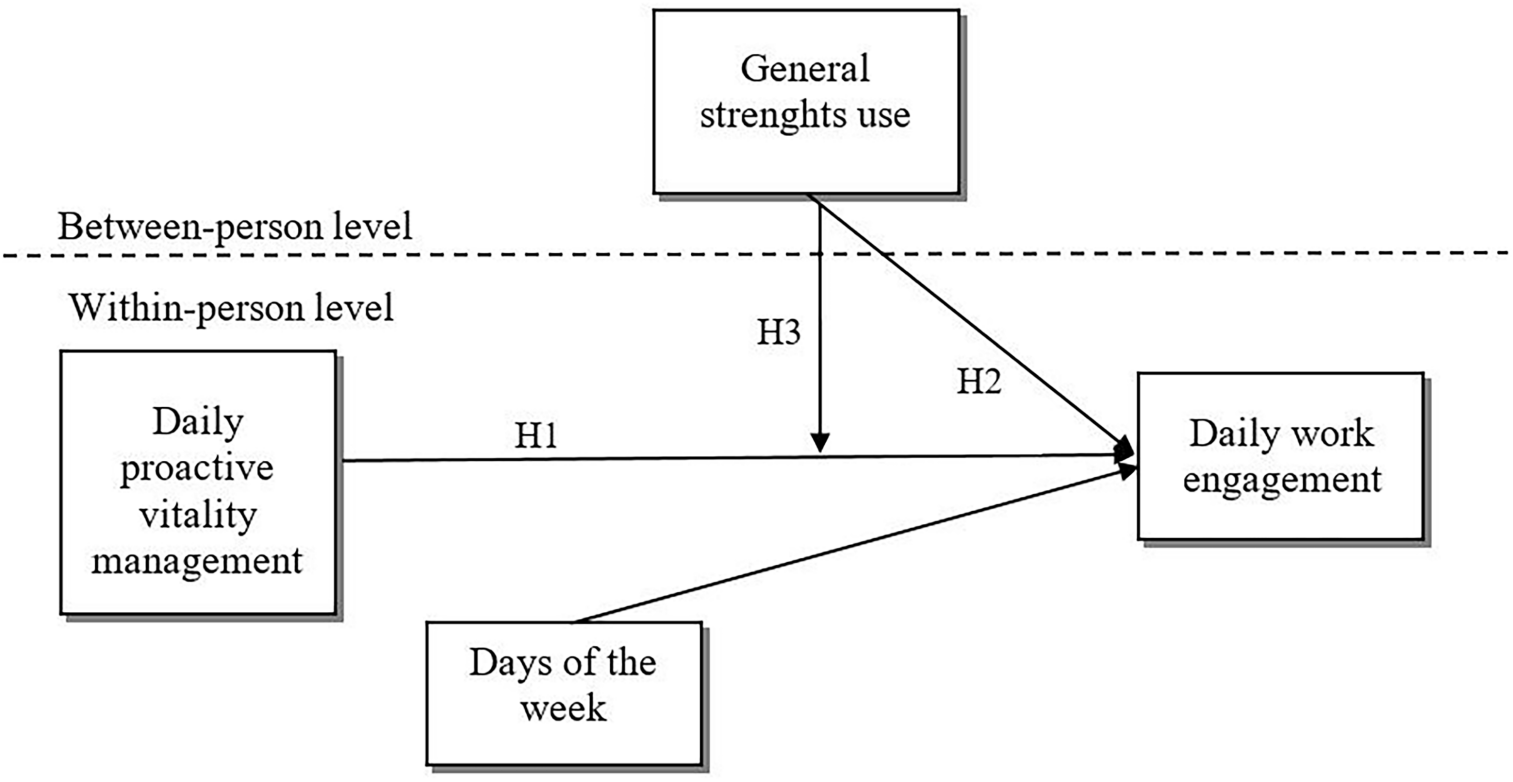
A multi-level model of work engagement, proactive vitality management, and strenghts use.

The proposed model (Figure 1) has its’ main contribution to the literature by testing interactions between bottom-up effects in the JD-R theory (Bakker and Demerouti, 2017). We argue that these bottom-up effects most likely do not operate independently from one another but having personal resources that ensure better alignment between the employee and the work, could enhance the efficiency of short-term, concrete behavioral strategies that contribute to maintaining a positive state of mind for work on a daily basis. Both the JD-R theory and the model of proactive motivation (Parker et al., 2010) have theorized that the efficiency of proactive initiatives can be enhanced or hindered by contextual variables, such as job resources and demands (i.e., job control, leader behavior), or individual-level factors such as goal orientation (Bakker et al., 2020) or self-insight (Op den Kamp et al., 2020). However, although personal resources could also manifest this effect from a theoretical perspective, there is no empirical work currently in the literature that would directly test this possibility.

Second, we highlight the relevance of proximal, hands-on strategies that employees themselves can implement daily to take personal initiative in improving their well-being. This moves forward from existing research which has for a long time focused on proactive actions aimed at changing aspects of the environment or the task (i.e., job crafting, Bakker, 2015) and much less on changing oneself (Parker et al., 2010). PVM is focused on the self (Op den Kamp et al., 2018), which makes it more accessible for employees daily, and less dependent on the work itself than enacting other forms of proactive behavior (i.e., renegotiating task boundaries).

Third, we assessed PVM and WE as time-varying constructs using a daily diary method. This approach minimizes retrospective bias by asking participants to refer to states and actions that have just occurred during their day (Bakker, 2014). Empirical evidence showed daily variations not only in WE (Sonnentag et al., 2010) but also in its’ antecedents, uncovering a dynamic and state-like experience of being engaged as a function of dynamic and state-like antecedents (Bakker, 2014). Bakker et al. (2020) were the first to link PVM to WE in a weekly design. However, while the authors have raised the issue that PVM is likely fluctuating also on shorter timeframes than a week, the research on this topic is relatively new and scarce. Therefore, this relationship has not yet been established at a day level.

### 1.1. Day-level relationship between daily proactive vitality management and daily work engagement

One main objective of the present study is to analyze the week-level positive relationship between PVM and WE (Bakker et al., 2020) at the day level. We argue that employees improve their daily engagement when they proactively manage their energetic, volatile, affective, and cognitive resources (Op den Kamp et al., 2018). Individuals can achieve this completely tailored to their personal, idiosyncratic needs and preferences, such as choosing to jog or walk to work to feel more physically energized and awake upon arrival or choosing to get comfortable in the car and play some inspiring or relaxing music to stay mindful and calm for an anticipated rushed workday (Op den Kamp et al., 2018). These strategies are not restricted to morning activities but can serve employees throughout the day. For example, if an employee has an important presentation in the middle of the day, engaging in PVM to optimize energy for that presentation could mean arranging other tasks earlier to gain a half-hour window to step outside and think through the presentation with a cup of coffee, or meditating before the presentation to get focused and present. The affective, energetic, and cognitive resources (i.e., task focus, optimism, positive affect) that are mobilized through PVM enable the employee to act and achieve objectives during the day (Sonnentag et al., 2010) and feel more vitality and engagement while working (Bakker, 2017). According to JD-R theory, such daily resource gain generated by PVM could significantly increase daily WE (Bakker, 2015; Bakker et al., 2020).

Empirical support for this assertion comes from related literature highlighting daily antecedents of WE in the form of positive affect, re-attachment to work, anticipated task focus (Sonnentag et al., 2020), and daily optimism (Tims et al., 2011). Bakker et al. (2020) empirically demonstrated a direct weekly relationship between PVM and WE. However, engagement is a state that also fluctuates in a shorter time frame of days (Bakker, 2014). PVM could represent a specific behavior through which an employee could achieve this state daily (Bakker and Demerouti, 2018). Based on this literature, we anticipate the following:

*H1*: Daily PVM is positively related to daily WE.

### 1.2. The cross-level effects of strengths use

Identifying key strengths is essential, but beyond the possession of certain strengths, the active use of these seems to be the key toward the most benefits in terms of reduced stress, more efficient work, increased self-esteem, vitality, and positive affect (Wood et al., 2011; van Woerkom et al., 2016). From the perspective of COR theory, this renders SU the role of a key personal resource (Ding and Lin, 2020; Chu et al., 2022), which can contribute to engagement by generating a resource caravan (Hobfoll, 2011). Research has shown that SU can facilitate engagement by employing personal strengths that match the job, increasing other resources such as person-job fit (Kooij et al., 2017), and self-efficacy and optimism (Meyers and van Woerkom, 2017; van Woerkom and Meyers, 2019). These, in turn, attract job resources in the form of developmental opportunities, positive feedback, or autonomy (Stander and Mostert, 2013). Employees’ initial investment of their strengths has the potential to draw a series of other resources that can also be invested further, creating a positive gain cycle (Hobfoll, 2011).

In a recent intervention study, Bakker and van Wingerden (2021) found that employees who learned to employ their strengths increased their WE. Following this empirical evidence, as well as other previous studies (van Woerkom et al., 2016; Bakker and van Woerkom, 2018), we propose that employees who generally use their strengths to complete their work will report increased daily engagement:

*H2*: SU at the individual level is positively related to daily WE.

Further, we argue that SU and PVM do not have only independent bottom-up relationships to WE, but rather, there could be a constant interaction between them. Within the JD-R theory, general personal dispositions can moderate the daily gain cycle between resources, WE, and proactive crafting behaviors (Bakker, 2015). Hence, individual differences, such as the propensity to engage in SU, might influence whether specific PVM strategies generate the desired effects or not (Bakker et al., 2020). Both JD-R and COR theories postulate that personal resources can have a significant role in the motivational gain cycle (Hobfoll, 2011) through their power to attract and gain other resources that maintain engagement. The enrichment of resources generated by SU as a personal resource (Chu et al., 2022) forms a resource caravan (Hobfoll, 2011) with the potential to create motivational states that sustain proactive action (Parker et al., 2010). Proactively engaging in vitality management can be easiest for an individual in activities that satisfy the conditions for proactive behavior to take place (Op den Kamp et al., 2018). Drawing on the proactivity model of Parker et al. (2010), we can expect that employees would engage in PVM with greater probability when they believe that they can have success in achieving their goal of getting into an energized state (“can-do motivation”), have some motivation to engage vigorously in the activity (“reason to motivation”), and when positive feelings activate them (“energized to”). The three states are fundamental in proactive goal generation and self-regulation to strive toward a self-initiated goal (Parker et al., 2010) and can be actively created by a personal resource such as SU. Empirical evidence supports this, showing that SU is linked to higher performance and proactivity (Miglianico et al., 2020; Tisu et al., 2022), as well as higher self-efficacy (van Woerkom et al., 2016). This suggests that employees who actively use their strengths build up performance coupled with beliefs in their success in work assignments where strengths have led to success. Other research shows that employees who achieve a better alignment between their strengths and their jobs can increase person-job fit, making the job more congruent with themselves and more personally motivating (Kooij et al., 2017). Other studies have provided results on the beneficial effects of SU in terms of positive affect and optimism (Meyers and van Woerkom, 2017).

While our study is the first to test the specific moderating effect of SU, recent research has provided empirical support for person-level moderators of the associations PVM has to well-being and performance. Op den Kamp et al. (2020) have found that PVM is more strongly related to creativity at the week level in the case of employees with higher self-insight (i.e., being more aware of ones’ states and feelings). In another study, Bakker et al. (2020) have shown that employees’ learning goal orientation moderates the weekly association between PVM and WE. Thus, we have formulated the following hypothesis:

*H3*: SU at the individual level enhances the daily positive relationship between PVM and WE.

## 2. Materials and methods

### 2.1. Participants and procedure

All 200 employees from a Romanian site of a multinational company were invited to participate in a study about well-being at the workplace voluntarily. This site functioned as a call center to offer support to clients in multiple languages across Europe. To our invitation, 104 employees enrolled in the study (52% response rate). We discarded data from 17 employees because they provided less than three daily responses, which has been suggested as a minimum number of observations needed to make inferences about daily relationships (Singer et al., 2003), leaving a total of 358 daily observations from 87 employees (44% response rate). The questionnaires were administered in web-based and paper-and-pencil forms, depending on employees’ access to online forms during work hours. In the first week of the study, participants completed a general questionnaire (demographic variables and SU). In the following week, they filled in the daily repeated measures. Employees received the daily questionnaire in the afternoon and had the rest of the entire working day to respond. None of the employees worked in shifts or weekends. Participants were offered an incentive to encourage daily participation. Participants who completed all measures were eligible to participate in the prize draw for an electric scooter offered up by the research team.

### 2.2. Sample characteristics

Participants (78.2% women) had a mean age of 30 years (SD = 4.76). The sample included employees in operational roles (69%), support functions (e.g., human resources, financial departments, 20%), and managers (11%). All participants worked full-time and had a permanent work contract. 17% of the participants were employed at the company for less than 1 year, 40% for over a year, and 43% for more than 2 years. 52% of the participants had a Bachelor’s degree, and 38% had a Master’s degree.

### 2.3. Measures

*Strengths use* was assessed by six items from the Strengths Use and Deficit Correction Questionnaire (van Woerkom et al., 2016), which has been previously translated and used in Romanian (Tisu et al., 2022). Participants rated their SU behavior (e.g., “I organize my job to suit my strong points”) on a 7-point Likert scale (0 = *almost never*, 6 = *almost always*). The scale had high internal consistency (α = 0.95).

*Daily proactive vitality management* was measured with eight items from the PVM scale (Op den Kamp et al., 2018), adapted to the Romanian context by Bălăceanu et al. (2022). Participants rated their PVM behaviors (e.g., “Today, I made sure that I could focus well on my work”) on a 7-point Likert-type scale (1 = *totally disagree*, 7 = *totally agree*). Cronbach’s alpha values were excellent across all measurements (0.87, 0.94, 0.92, 0.95, 0.94, with a mean of 0.92).

*Daily work engagement* was measured with six items from the Utrecht Work Engagement Scale (UWES-9; Schaufeli et al., 2006), adapted to the Romanian context by Vîrgă et al. (2009). Following Bakker and Xanthopoulou (2009), we considered two items for each of the three dimensions: vigor (“Today, I was bursting with energy while working”), dedication (“Today, I was enthusiastic about my job”), and absorption (“Today, I got carried away when I was working”). Responses were given on a 7-point scale (0 = *completely disagree*, 6 = *completely agree*). The scale had good reliability across the five days (0.77, 0.89, 0.87, 0.87, and 0.86, with a mean of 0.85).

### 2.4. Construct validity

To establish multilevel construct validity, we conducted multilevel confirmatory factor analyses (MCFA) using MPlus 8 (Muthén and Muthén, 1998). Goodness-of-fit was evaluated using the χ2 likelihood ratio statistic alongside the comparative fit index (CFI), the Tucker–Lewis Index (TLI), the root mean square error of approximation (RMSEA), and the standardized root means square residual (SRMR). Values of 0.90 or higher for CFI and TLI, and 0.08 or lower for RMSEA and SRMR indicate an acceptable model fit to the data (Hu and Bentler, 1999). Models were compared using the Satorra-Bentler scaled chi-squared difference (Satorra and Bentler, 2010) and the difference in CFI, with ΔCFI >0.01, indicating a significant change in model fit (Cheung and Rensvold, 2002).

Table 1 contains the results of the MCFA. The hypothesized 3-factor model (M1) placed SU at Level 2 (L2) and defined PVM and WE as distinct but correlated Level 1 (L1) factors. This model had a good fit to the data [χ^2^(106) = 3131.83, *p* < 0.001; CFI = 0.92; TLI = 0.9; RMSEA = 0.08; SRMR_within_ = 0.07; SRMR_between_ = 0.03]. To verify if PVM and WE are indeed best conceptualized as within-person factors, we compared this model with a 5-factor model (M2) in which we defined latent factors for PVM and WE on both levels. This model faired significantly worse [χ^2^(281) = 3537.15, *p* < 0.001; CFI = 0.71; TLI = 0.68; RMSEA = 0.09; SRMR_within_ = 0.18; SRMR_between_ = 0.23; Δχ^2^(15) = 86.16, *p* < 0.001; ΔCFI = 0.21]. This comparison assured that PVM and WE are better conceptualized and modeled only at L1. To further test the discriminant validity of the L1 measures, we also compared the hypothesized model to a 2-factor solution (M3), which merged PVM and WE in one factor at L1. M3 also performed significantly worse compared to M1 [χ^2^(106) = 3131.83, *p* < 0.001; CFI = 0.81; TLI = 0.76; RMSEA = 0.16; SRMR_within_ = 0.07; SRMR_between_ = 0.03; Δχ^2^(3) = 187.58, *p* < 0.001; ΔCFI = 0.11]. This comparison provides support for the conceptual distinctiveness of PVM and WE. Lastly, to test for the risk of common method bias, we made the last comparison to a model which defined only one latent factor at L2 for all items (M4). M4 performed unacceptably on all indices [χ^2^(281) = 3537.15, *p* < 0.001, CFI = 0.22; TLI = 0.20; RMSEA = 0.15; SRMR_within_ = 0.38; SRMR_between_ = 0.31].

**Table 1:** Fit indices from the multilevel confirmatory factor analysis.

Model	*χ* ^2^ (df)	*Δχ* ^2^ (Δdf)	CFI	ΔCFI	TLI	RMSEA	SRMR_Within_	SRMR_Between_
M1–3 factors	3131.83 (106)^***^		0.92		0.90	0.08	0.07	0.03
M2–5 factors	3537.15 (281)^***^	86.16 (15)^***^	0.71	0.21	0.68	0.09	0.18	0.23
M3–2 factors (common latent factor at L1)	3131.83 (106)^***^	187.58 (3)^***^	0.81	0.11	0.76	0.16	0.07	0.03
M4–1 common latent factor at L2	3537.15 (281)^***^	194.51 (3)^***^	0.22	0.7	0. 20	0.15	0.38	0.31

*N* = 421; Δχ^2^, Satorra-Bentler scaled chi-squared difference based on the log-likelihoods; df, degrees of freedom; CFI, comparative fit index; TLI, Tucker-Lewis index; RMSEA, root mean square error of approximation; SRMR, standardized root mean square residual. Each alternative model was compared to M1 (hypothesized 3-factor model with WE and PVM as L1 factors, and SU as L2 factor). ^***^p < 0.001.

### 2.5. Hypotheses testing

We applied Hierarchical Linear Modeling using Maximum Likelihood estimation with robust standard errors in MPlus 8 to test the proposed model (Muthén and Muthén, 1998). This approach entails separating the variability at the within-person and between-person levels to test both intra-individual variations and inter-individual differences (Singer et al., 2003). In the first step, we assessed intercept variability in WE. In the next step, we regressed WE on time in the form of days of the week (centered around the first measurement point) and on PVM (centered around the person-mean). In the third step, we allowed the slopes of the relationship between WE and PVM to vary across employees. In the last step, we tested the cross-level effects of SU by regressing the intercept and the PVM – WE slope on SU, centered around the grand mean. We performed simple slope tests using Preacher’s online tool for a detailed analysis of the cross-level interaction (Preacher et al., 2006). After each step, we calculated pseudo-R^2^ on the total-, within-, and between-variance, tested the improvement in model fit using the Akaike Information Criteria (AIC), Bayesian Information Criteria (BIC), and calculated a difference test using the Satorra-Bentler scaled chi-square, based on the log-likelihoods (Satorra and Bentler, 2010).

## 3. Results

Zero-order correlations, means, standard deviations, and scale reliabilities are summarized in Table 2. Attrition varied between 10.3% (on day 1) and 28.7% (on day 5). Additionally, we calculated a multilevel correlation among the daily measures, which showed a significant within-person association between PVM and WE (Estimate = 0.59, *p* < 0.001).

**Table 2:** Descriptive statistics, scale reliabilities, and bivariate correlations among daily PVM, WE, and general SU.

M (SD)		α	Day 1 PVM	Day 2 PVM	Day 3 PVM	Day 4 PVM	Day 5 PVM	Day 1 WE	Day 2 WE	Day 3 WE	Day 4 WE	Day 5 WE
SU	5.31 (1.28)	0.95	0.37^**^	0.28^*^	0.19	0.27^*^	0.28^*^	0.30^*^	0.28*	0.15	0.11	0.28^*^
Day 1 PVM	3.88 (0.49)		0.87	0.67^***^	0.55^***^	0.53^***^	0.53^***^	0.67^***^	0.51^***^	0.44^***^	0.50^***^	0.35^**^
Day 2 PVM	3.77 (0.70)			0.94	0.81^***^	0.65^***^	0.45^**^	0.56^***^	0.76^***^	0.64^***^	0.48^***^	0.41^**^
Day 3 PVM	3.75 (0.62)				0.92	0.64^***^	0.54^***^	0.53^***^	0.68^***^	0.79^***^	0.58^***^	0.49^***^
Day 4 PVM	3.71 (0.76)					0.95	0.69^***^	0.46^***^	0.66^***^	0.58^***^	0.75^***^	0.64^***^
Day 5 PVM	3.79 (0.62)						0.94	0.44^**^	0.49^***^	0.53^***^	0.59^***^	0.81^***^
Day 1 WE	3.08 (0.60)							0.77	0.69^***^	0.63^***^	0.60^***^	0.40^**^
Day 2 WE	3.13 (0.78)								0.89	0.80^***^	0.74^***^	0.60^***^
Day 3 WE	3.19 (0.75)									0.87	0.66^***^	0.64^***^
Day 4 WE	3.16 (0.75)										0.86	0.71^***^
Day 5 WE	3.19 (0.72)											0.85

***p < 0.001, **p < 0.01, *p < 0.05, Cronbach’s α coefficients are displayed on the main diagonal. N_day1_ = 78, N_day2_ = 75, N_day3_ = 76, N_day4_ = 67, N_day5_ = 62.

Table 3 reports the main analysis results in each model-building step. A significant chi-squared difference between models, alongside a progressive decrease in AIC and BIC values, indicated substantial improvement in model fit after each step in the analysis. WE showed an intra-class correlation (ICC) of 0.622, and PVM showed an ICC of 0.577, suggesting that 62%, respectively, 57% of the variance in these constructs can be explained by inter-individual differences. Conversely, 38% of the variance in WE and 43% in PVM is intra-individual and can be explained by within-person changes over the week.

**Table 3:** Model results.

Level and variable	Null model	Fixed L1 predictors	Random slopes	Cross-level effects
*Level 1*				
Intercept	3.16^***^ (0.06)	3.08^***^ (0.08)	3.01^***^ (0.07)	3.01^***^ (0.07)
Day (γ_10_)		0.04^**^ (0.02)	0.03^*^ (0.02)	0.04^*^ (0.02)
PVM (γ_20_)		0.64^***^ (0.07)	0.68^***^ (0.06)	0.67^***^(0.06)
*Level 2*				
SU (γ_01_)				0.12^*^(0.05)
SU*PVM (γ_11_)				0.14^**^(0.05)
*Variance components*				
L1 variance	0.19^***^(0.03)	0.12^***^(0.02)	0.11^***^(0.02)	0.10^***^(0.02)
L2 variance	0.31^**^ (0.06)	0.32^***^ (0.06)	0.33^***^ (0.06)	0.30^***^ (0.05)
Slope variance (μ_1j_)			0.08^**^ (0.03)	*0*.*05 (0*.*03)*
Intercept-slope covariance (σ_μ01_)			0.08^*^ (0.03)	*0*.*06 (0*.*03)*
ICC	0.62			
*Model fit information*				
∆AIC		−122.96	−9.39	−7.04
∆BIC		−121.55	−7.97	−5.62
-2LL (df)		96.63 (2)^***^	33.89 (2)^***^	10.33 (2)^**^
Number of free parameters	3	5	7	9
Pseudo R^2^ total		0.11 (11.3%)	0.02 (2.3%)	0.06 (5.8%)
Pseudo R^2^ within		0.38 (37.6%)	0.11 (11%)	–
Pseudo R^2^ between		–	–	0.08 (7.7%)

L1, inter-individual level 1; L2, intra-individual level 2; SU, strengths use; PVM, proactive vitality management; WE, work engagement; Robust standard errors of estimates are in parentheses. ***Significant at *p* ≤ 0.001; **Significant at *p* ≤ 0.01; *Significant at *p* ≤ 0.05; italicized estimates are non-significant.

The results showed that the weekday variation significantly and positively predicted WE (γ_10_ = 0.04, SE = 0.02, *p* = 0.02), indicating that employees became slightly more engaged as the week progressed. In line with the first hypothesis (H1), PVM positively predicted WE (γ_20_ = 0.64, SE = 0.06, *p* < 0.001), showing that employees were more engaged during the days when they more proactively managed their vitality. Variability in the workdays and PVM explained 11.3% of the total variance of WE. There was also a significant intercept-slope covariance (σ_μ01_ = 0.08, SE = 0.03, *p* = 0.02), indicating that individuals who started the week at a higher level of WE experienced a stronger daily relationship between PVM and WE. Furthermore, the PVM-WE slope showed significant inter-individual variability (μ_1j_ = 0.08, SE = 0.03, *p* = 0.005), suggesting that the positive relationship between PVM and WE varies across individuals, and inter-individual differences might explain its’ variation. Allowing random slopes explained an additional 2.3% of the total variance in WE.

**Figure 2:**
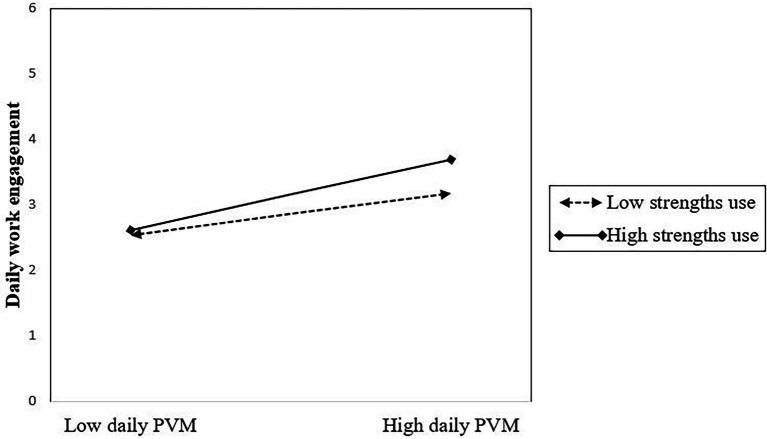
The cross-level interaction between general SU and daily PVM in predicting daily WE.

The second hypothesis (H2) postulated a significant direct cross-level effect of SU on the WE intercept. The data showed that higher levels of general SU reported in the first week of the study predicted a higher average of daily WE in the following week (γ_01_ = 0.12, SE = 0.05, *p* = 0.012). Furthermore, SU significantly predicted the PVM – WE slope (γ_11_ = 0.14, *p* = 0.006), which supports our third hypothesis (H3), postulating a cross-level moderating effect of SU. Adding SU to the model explained an additional 6.5% of the total variance and 8.3% of the between-person variability in WE. The simple slope analysis showed that at lower levels of SU (1 SD below the mean), PVM predicted WE positively (β = 0.49, SE = 0.09, *p* < 0.001), but this relationship became significantly stronger (*t* = 2.9, df = 712, *p* = 0.003) at higher levels of SU (+1 SD above the mean; β = 0.85, SE = 0.08, *p* < 0.001). This means that the strongest benefits of daily PVM in terms of increased engagement could be observed in the case of those employees who also generally relied more on their strengths to organize and complete their tasks (Figure 2).

## 4. Discussion

This research adopted a diary method to investigate the daily relationship between PVM and WE throughout a workweek. Based on previous research (Op den Kamp et al., 2018; Bakker et al., 2020), we expected to find a dynamic daily relationship between PVM and WE that inter-individual differences in SU can moderate.

The data showed significant variability and a slight increase in WE from the beginning to the end of the workweek, replicating previous findings regarding daily changes in employee engagement (Sonnentag et al., 2010, 2020; Bakker, 2014). As predicted by H1, on days when employees actively managed their cognitive, emotional, and energetic resources, they also reported feeling more engaged in their work. These results are aligned with previous research demonstrating relationships between PVM and WE (Op den Kamp et al., 2018; Bakker et al., 2020; Bălăceanu et al., 2022; Tisu et al., 2021). From the perspective of the JD-R theory (Bakker and Demerouti, 2017), PVM represents a specific type of self-regulatory behavior, which, similar to job crafting (Tims and Bakker, 2010), can contribute to the motivational process through the resource gains that are generated by engaging in the behavior. While PVM differentiates from job crafting through the distinct focus on the self instead of aspects of the work, it can work through a similar process, actively impacting the resources (especially energetic and affective resources) that employees then draw from to stay engaged in work tasks (Op den Kamp et al., 2018). For example, an employee can decide to take the bike to work in the morning, with the proactive goal of arriving in a more energized state for a morning task. Another employee might meditate shortly before starting a difficult task to create a more absorbed state in the activity.

The most important findings of the present research reside in the significant cross-level direct (H2) and moderating (H3) effects of SU. These results are aligned with previous cross-sectional and longitudinal research (van Woerkom et al., 2016; Bakker and van Woerkom, 2018). Existing studies focusing on the outcomes of SU have shown that relying more on strengths to complete work increases person-job fit (Kooij et al., 2017), boosts personal resources, and positive affect (Meyers and van Woerkom, 2017; van Woerkom and Meyers, 2019), attributing SU the role of a personal resource, which attracts and generates other resources that an individual needs to invest to achieve high levels of well-being (Hobfoll, 2011). JD-R theory’s perspective conveys that SU has an essential moderating role in the motivational gain cycle (Bakker, 2015). Aligned with these theoretical frames, the proactivity literature (Parker et al., 2010) and previous studies investigating SU as a personal resource (Ding and Lin, 2020; Chu et al., 2022), the data confirmed our expectations that PVM would predict the highest engagement in the case of employees who direct their energy and effort toward activities they use their key strengths in. This could be because the resources and states associated with SU facilitate the motivational states necessary for mobilizing and sustaining proactive action (Parker et al., 2010). By creating the conditions in which proactive goal generation and behavior thrive, SU seems to be an essential person-level catalyst for the benefits of PVM. For example, suppose an employee is highly creative and uses this strength frequently to perform his/her work over time. In this case, tasks that can imply creativity can be associated with feelings of competence, self-efficacy, and anticipated positive affect. These can prompt and maintain proactive initiatives toward activities that energize the employee (e.g., taking a walk outside to get fresh air) or facilitate a focused state (e.g., meditating, researching other creative works that are connected) whenever the employee anticipates that using his/her creativity in the task will be possible and beneficial.

### 4.1. Theoretical implications

In this research, we adopted a bottom-up, employee-focused perspective, highlighting proactive behaviors and resources that employees bring into the well-being dynamics in organizations. Concretely, we found that daily engagement can be achieved by employees’ active and proactive generation of positive psychological states and their efforts to make the most of psychological resources. This focus complements top-down approaches that scholars and employers generally take to promote WE in organizations (Bakker, 2014; Op den Kamp et al., 2018) by highlighting that employees have a proactive influence over their well-being and are not only passive receivers of traditionally researched top-down effects (Bakker and Demerouti, 2018). Moreover, following previous studies proposing that the efficiency of PVM can be moderated by stable person-level and environment-level factors (Bakker et al., 2020; Op den Kamp et al., 2020), we showed that personal resources that also imply a propensity toward proactive action (such as SU), could improve the daily positive effects of PVM. This means that the bottom-up effects enlisted within the JD-R theory (Bakker, 2017) do not exist and operate only independently. Rather, our theoretical understanding of them can be expanded if we also consider that short-term, concrete behaviors work in interaction with stable proactive personal resources.

### 4.2. Practical implications

In terms of practical insights, this research suggests that creating awareness around such proactive strategies and allowing employees the freedom and opportunity to engage in them could return high levels of daily engagement. Beyond encouraging employees to use their strengths and manage their vitality, organizations also can take action through training interventions facilitating PVM and SU. Organizations that wish to promote such behaviors through training can build on valuable recent findings. The results of a recent intervention study showed that training based on energy management techniques led to increases in PVM (Bălăceanu and Vîrgă, 2022). Helping employees develop PVM strategies and allowing them time and space to implement these consistently across the week could greatly benefit maintaining high energy levels throughout the week. In the after-COVID context, with work from home isolated from the resources that the presence of colleagues and managers offer during the day (van Zoonen and Sivunen, 2021) and different plans of returning to work that heavily relies on remote participation, PVM can become a proper individual strategy for employees who become more personally responsible than ever for staying focused and engaged during the day.

Similarly, SU interventions gained scientific terrain in recent years (Miglianico et al., 2020). A recent meta-analysis investigating the effectiveness of interventions targeting strengths identification, development, and use in organizations shows that such interventions generate moderate increases in well-being, slight increases in proactive personal strategies, and strong growth of personal resources (Vîrgă et al., 2022). Our results underline that when employees create opportunities for SU, they not only reap the direct benefits of this strategy but also gain more from their PVM. This suggests that a complementary development of these behavioral strategies could benefit employees the most.

### 4.3. Strengths, limitations, and future research

A significant strength of our research resides in the diary design, which allows a more naturalistic investigation of the relationships to WE, minimizing the risks of recall bias and capturing short-term reports close to the reality of everyday working life (Sonnentag et al., 2010, 2020). Regarding limitations, first, we collected self-report data, which raises concerns about common method bias. To minimize this risk, we have carefully analyzed and compared the proposed multilevel factor structure to solutions where measurements overlapped in a common latent factor and assured that the proposed model represented the data best. Future research could obtain data from different sources (e.g., supervisors’ ratings) to rule out other sources of common method bias (Podsakoff et al., 2003).

Second, the sample size at the employee level might limit our conclusions’ robustness regarding the random and cross-level effects. L2 sample sizes greater than 30 tend to have a minimal impact on the accuracy of the fixed effects. However, recommendations for L2 units necessary for computing accurate standard errors of variance components range from 30 units to over 100 (Scherbaum and Ferreter, 2009). We also draw a cautionary note on the generalizability of these findings to the larger working population. Collecting the data within one organization contributes to the internal validity of our research, but it comes at the cost of external validity. Considering this, our findings apply primarily to highly educated white-collar female workers. Using this model in other work contexts could be inadequate because such proactive behaviors could have different boundary conditions and forms of manifestation in other contexts. Thus, knowing that PVM can contribute to daily engagement, it becomes essential that future research uncovers the structural and contextual antecedents and conditions of such behaviors. This could inform managers and HR practitioners about working conditions that impede or facilitate such proactive behaviors beyond the employee’s initiative. For example, Van Scheppingen et al. (2015) have found that vitality at work was positively associated with a balanced orientation toward work and social capital at the workplace. Op den Kamp et al. (2020) have found that, alongside self-insight, social support at the workplace was a significant moderator of the positive effects of PVM. In the original validation study of the PVM concept (Op den Kamp et al., 2018), the authors highlighted that employees enjoying high levels of autonomy and skill variety might have more opportunities to engage in vitality management strategies, which might not be accessible in all occupations yet. However, they also pointed out that since these strategies are profoundly personal and can be tailored to anyone’s preferences and context, workers across all industries and occupations can benefit from them with some progress in supporting their use. Therefore, replicating our results with a more extensive sample of individual employees and explicitly testing potential differences between industries and occupations could further our knowledge not only about PVM as a concept but also about the contexts which favor its’ manifestation.

Finally, although the sequence we tested in this study from PVM to WE is based on theory and earlier research, other orderings are also plausible. It is also possible for WE to further predict PVM, similar to the daily cycles through which WE feed back into job crafting behaviors (Tims and Bakker, 2010; Bakker, 2015). Future research investigating gain cycles is an important and necessary development in deepening our current understanding of the dynamics of WE and proactivity in organizations (Bakker and Demerouti, 2018). Similarly, our model is aligned with the predominant discussions within the JD-R literature, emphasizing that personal resources can attract and protect other resources (Bakker and Demerouti, 2017). However, some authors also stress a bidirectional, interactive relationship between resources and personal resources. For example, Kira et al. (2010) have discussed ways in which collaborative job design (i.e., job characteristics and resources shaped in close collaboration with employees and their needs) can support the development of personal resources. This potential reversed, and bidirectional causation is fundamental in our understanding of the modern world of work. Therefore, it creates exciting new directions for future research on proactivity in the workplace.

## 5. Conclusion

The current study showed that employees who proactively manage their physical and mental energy and use their key strengths to complete work report the highest daily engagement. Through PVM, employees actively manage their cognitive, emotional, and energetic resources and increase their engagement throughout the workday. Moreover, employees who rely on their key strengths to complete their work feel more engaged and benefit more from PVM. Thus, our results demonstrate that while proactive strategies are beneficial independently, they yield the most gains when combined and used complementary.

## Data availability statement

The raw data supporting the conclusions of this article will be made available by the authors, without undue reservation.

## Ethics statement

Ethical review and approval were not required for the study on human participants in accordance with the local legislation and institutional requirements. Written informed consent to participate in this study was provided by the participants’ legal guardian/next of kin.

## Author contributions

ZP and DV contributed to the design, choice of theories, and elaboration of hypotheses. DV and DL collected and cleaned the data. DV contributed to constructing arguments and coordinated the writing process. ZP did the analyses and produced the manuscript. All authors contributed to the article and approved the submitted version.

## Funding

The work of ZP and DV was supported by a grant from the Ministry of Research, Innovation and Digitization, CNCS/CCCDI – UEFISCDI Romania, project number PN-III-P4-ID-PCE-2020-1880, within PNCDI III (https://uefiscdi.gov.ro/).

## Conflict of interest

The authors declare that the research was conducted in the absence of any commercial or financial relationships that could be construed as a potential conflict of interest.

## Publisher’s note

All claims expressed in this article are solely those of the authors and do not necessarily represent those of their affiliated organizations, or those of the publisher, the editors and the reviewers. Any product that may be evaluated in this article, or claim that may be made by its manufacturer, is not guaranteed or endorsed by the publisher.
